# Peach Palm (*Bactris gasipaes* Kunth.): Ancestral Tropical Staple with Future Potential

**DOI:** 10.3390/plants11223134

**Published:** 2022-11-16

**Authors:** Nancy González-Jaramillo, Natalia Bailon-Moscoso, Rodrigo Duarte-Casar, Juan Carlos Romero-Benavides

**Affiliations:** 1 Maestría en Alimentos, Facultad de Ciencias Exactas y Naturales, Universidad Técnica Particular de Loja, Loja 110108, Ecuador; 2 Departamento de Química, Facultad de Ciencias Exactas y Naturales, Universidad Técnica Particular de Loja, Loja 110108, Ecuador; 3 Facultad de Ciencias de la Salud, Universidad Técnica Particular de Loja, Loja 110108, Ecuador; 4 Departamento de Turismo y Gastronomía, Facultad de Ciencias Administrativas y Económicas, Universidad Técnica de Manabí, Portoviejo 130105, Ecuador

**Keywords:** palm, *Bactris gasipaes*, food sovereignty, Amazon, phytochemicals

## Abstract

A pre-Columbian staple, *Bactris gasipaes* Kunth. is a palm tree domesticated around 4000 years ago, so appreciated that a Spanish chronicler wrote in 1545, “only their wives and children were held in higher regard” by the Mesoamerican natives. The peach palm is an integral part of the foodways and gastronomy of Ecuador, Colombia, Bolivia, Peru, Brazil, and other tropical American countries; meanwhile, it is almost unknown in the rest of the world, except for hearts of palm. Although abundant, the species faces anthropogenic threats. The purpose of this study is to describe and summarize the physicochemical, nutritional, and bioactive characteristics of the peach palm and its two main alimentary products: hearts of palm and fruits, highlighting the functional and antioxidant potential of the latter, showing both ancestral and modern uses. There is active research on peach palm products and coproducts that aim for better, more sustainable uses of its traditional and recently found properties. The review and presentation of studies on this strategically relevant species can motivate the protection of endangered populations and stimulate new lines of research to advance development in the food, pharmaceutical, and cosmetic industries, with fair trade, sustainable development goals, and adaptation to climate change in mind.

## 1. Introduction

The Amazon is a multicultural and multiethnic space where natural resources have been used ancestrally to provide housing, medicine, and food to its inhabitants [1]. Some of these are fruits that are currently considered promising for their sensory, nutritional, and ethnomedicinal characteristics [2,3,4]. Among these important species, palms are an important family. Palm trees are considered to be more useful to mankind than any other family, inspiring the tree of life motif [5]. An important palm in the Amazon and tropical America is *Bactris gasipaes* Kunth., in the *Areacaceae* family, the most important pre-Columbian American palm [6], a polyvalent pre-Columbian staple. This species, among many others, is expected to undergo changes in the land area suitable for cultivation due to climate change, with loss of current cultivation areas and the emergence of new ones [7]. This would not be the first climate event in Amazonia [8], and *B. gasipaes* overcame earlier events.

There is abundant research published on *B. gasipaes,* including excellent review articles [9,10]. We found the need for an integrated, updated view that presents the current knowledge of the food and phytochemical study of the species. Most research on the species centers on its biology and agriculture.

This article discusses the ancestral and modern uses of *B. gasipaes*: food, pharmacological, and others, with emphasis on the fruit of the species. We aim to find research and application opportunities in food and industrial production in the face of the food crisis that favors a return to more ecological agroecosystems [11]. We also provide material for action on the following Sustainable Development Goals (SDG): 1 No poverty, 2 Zero hunger, 9 Industry, innovation and infrastructure, 12 Responsible consumption and production, 13 Climate action, and 15 Life on land.

## 2. Methods

A narrative literature review was performed consulting the published literature in English, Spanish, and Portuguese for “*Bactris gasipaes*” in the title, abstract, and keywords; the last two languages were included because they are the main languages spoken in the countries where the species grows. The scientific databases consulted were Scopus (413 results), Web of Science (218), Crossref (494), Dimensions (505), and SciELO (136). Among the documents returned by the searches, those having to do with history, domestication, ancestral and modern food, and feed uses, ethnopharmacology, cultural aspects, phytochemical composition, sustainability, and innovation were included in the review. Results from the last fifty years were included in the search. Ad hoc searches were performed as needed to supplement the information and relevant, earlier publications are also mentioned.

## 3. Taxonomy and Distribution

*Bactris gasipaes* Kunth. Belongs to the *Bactris* Jacq. ex Scop. genus of spiny palms in the *Arecaceae* family, *Cocoseae* tribe. The genus includes seventy-nine species, of which several are edible. *B. gasipaes* is the most used species as a food resource in the genus. There are two accepted varieties of the species: *Bactris gasipaes* var. *chichagui* (H. Karst.) A.J. Hend. [12], and *Bactris gasipaes* var. *gasipaes* [13,14]. The *chichagui* variety is mostly wild with smaller, oilier fruit, and the *gasipaes* variety is cultivated, with larger and starchier fruit [15].

*B. gasipaes* currently extends throughout the Amazon basin and other humid lowlands of the neotropics, with one population up to 1800 m above sea level in Colombia. The species has been introduced to countries such as Australia, Indonesia, Malaysia, Reunion island, and Hawaii [16,17,18] to relieve the pressure on local palms endangered by non-sustainable production, especially where hearts of palm are produced.

**Figure 1:**
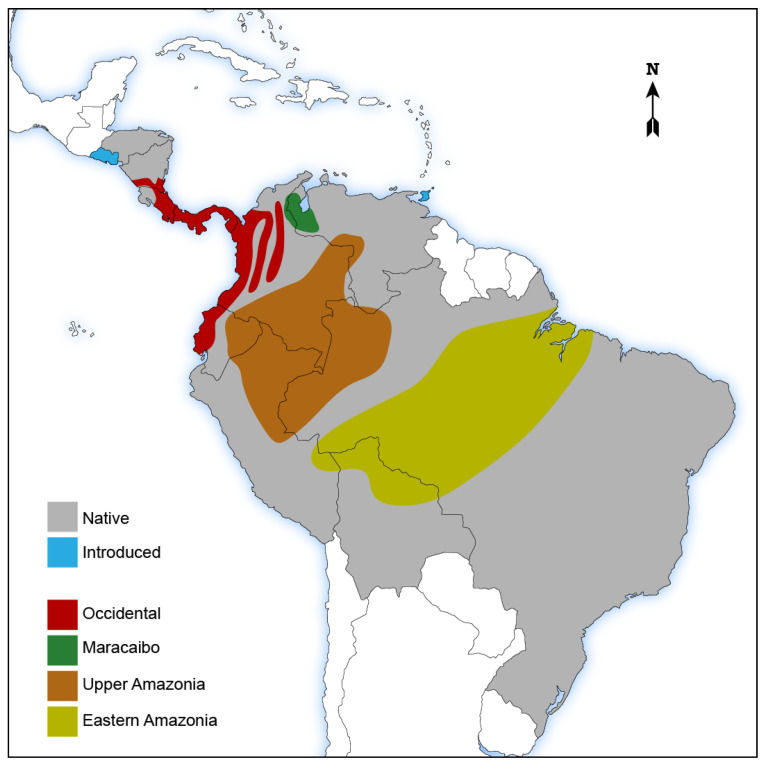
Distribution of *Bactris gasipaes* Kunth., in tropical Central and South America. Grey: native; light blue: introduced (Trinidad and Tobago, El Salvador). Source: [19]. Shaded areas, main complexes: red: Occidental; green: Maracaibo; brown: Upper Amazonia; yellow: Eastern Amazonia. Source: [20].

Common names for this species vary depending on the region, some of which are presented in Table 1. Figure 1 shows the distribution of *B. gasipaes* in tropical America by country, both as a native and as an introduced species.

## 4. Morphological Description

**Figure 2:**
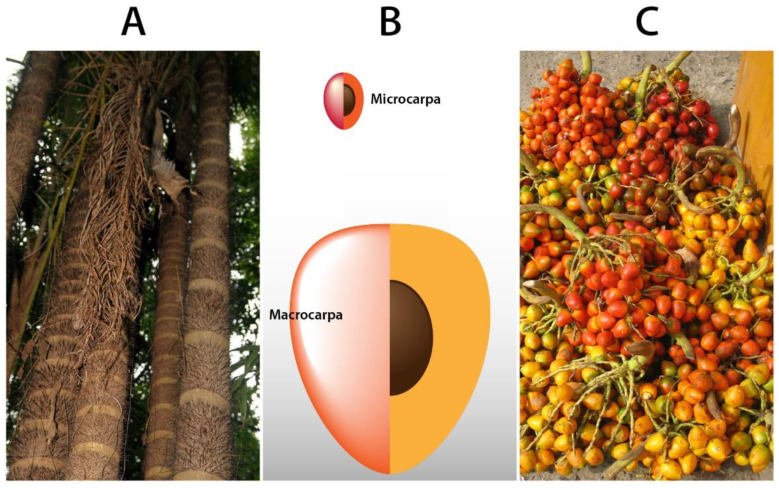
Morphology of *Bactris gasipaes*: (**A**). Trunks showing internodes covered with thorns. Source: David Stang (CC BY-SA 4.0); (**B**). Difference between macrocarpa and microcarpa fruits; (**C**). *B. gasipaes* fruit, microcarpa variety, Cali Colombia. Source: Michael Hermann (CC BY-SA 3.0).

The species is characterized by being cespitose, i.e., several trunks (one to fifteen) with heights from six to twenty meters stemming from the point of origin, smooth in appearance, with evident internodes, surrounded by a ring of black thorns (Figure 2A) [16,20]. The leaves, located at the top of the trunk, are arched and long (up to 100 cm), their sheath covered with small white or dark brown spines; inflorescences appear in intrafoliar clusters and are monoecious: female and male flowers emerge from the same stem; with some structures, such as the peduncle, covered with spines. The species can produce up to ten inflorescences per year [17,21].

Inside the trunk, the heart of palm can be found, a vegetable considered a delicacy: cylindrical and sweet-tasting, it consists of the apical meristem, divided into basal, central and apical with marked differences in texture due to the arrangement of the structural fibers; its harvest results in the death of some species [22,23], but not in *Bactris* spp., and other cespitose palms. This is why it has become a more sustainable prime source for hearts of palm, together with *Euterpe oleracea* (Açai).

The fruits are spherical or ovoid in shape, and come in a variety of sizes; the largest are up to10 cm long and up to 6 cm in diameter, they are clustered, green when unripe, and from yellow to orange when ripe [5,24]. The inside of the pulp is floury or oily depending on the variety, and in the center, there is a seed that resembles a very oily small coconut (Figure 2B). According to the weight of the fruit, *B. gasipaes* is categorized as *microcarpa* (<20 g), *mesocarpa* (>21 g, <70 g) and *macrocarpa* (>70 g) (Figure 2C), and also as orange, yellow, and lately, white varieties [25,26]. The fruit presents low polyphenol oxidase activity, which makes it suitable for minimally processed products [18].

The similarity with other species suggests the species was domesticated in areas of the Amazon through hybridization [5,27,28]. Its food use is mainly for its oil content, plus the harvesting process has ethnic, cultural, and traditional value [5,15,28,29]; in addition, it is attractive as a promising industrial resource for new products derived from the same species (chonta, palm heart, wood) [30,31].

## 5. Mythical Origins

There are several related origin myths for *B. gasipaes* in the traditions of Peruvian (Asháninka), Colombian (Yukuna Matapi), and Ecuadorian (Shuar Achuar) native peoples [32,33,34,35]. A common motif is that chonta and maize come from the otherworld, stolen by human or demigod visitors. In a Catío myth, the immortals of *Armucurá*, a planet below earth, feed on the vapors of *B. gasipaes*, but it is the human visitors that return with seeds for human cultivation and consumption [35]. In these stories, practical elements are transmitted: the quality of the wood for the making of proper weapons; the food value of the species; and the importance of not throwing the seeds away but saving them for cultivation. The interdependence of the cultivated palm and humans is shown in these stories, pointing to a domesticated species, no longer able to survive in the wild. Another example of how ingrained *B. gasipaes* is in the local cultures is the calendar use: “Pupunha summer” is part of the calendar of the indigenous peoples of the Tiquié river in Brazil [36], and the harvest season between December and March is an important event. Today, chonta festivities that reenact these myths survive and are becoming a community tourism product [37].

## 6. History

The origin of the species is unclear, but consensus points to the Bolivian amazon as a possible origin [5]. Three hypotheses coexist: a southwestern Amazon domestication, a northwestern South America domestication, and that of multiple origins. Two dispersals are supported by genetic diversity patterns: one along the Ucayali river, northwestern South America, and into Central America with a starchy, fermentable fruit used as a staple; another, along the Madeira river into central and eastern Amazonia in which the smaller, starchy, and oily fruit is used for snacking [15]. Similarity with other species suggests the species was domesticated in areas of the Amazon through hybridization, with highest genetical diversity in northern Peruvian and Ecuadorian Amazon. The domestication of the species is tentatively dated between 4000 and 3000 years BP, coincident with the establishment of sedentary communities in Amazonia [5,27,28]. The earliest archaeological remains of the species, presumably already cultivated, are dated 2300–1700 BC in Costa Rica [38].

The records about the species date back to the fifteenth century, and it is presumed that the beginning of its production under the Spanish was as timber, not as a food resource. During the Spanish conquest, native crops such as *B. gasipaes* lost importance, due to the massive native population loss [8] and the adoption of European productive systems. The European contact in the fifteenth–sixteenth centuries in Central America is marked by the awe of the Spaniards towards how appreciated the species was. Spanish chronicler Godínez Osorio wrote in 1575 that, “only their wives and children were held in higher regard” [39]. The timber of *B. gasipaes* was used in the early 1540s by the Spaniards to build fortifications because of its hardness and the fact that the trunk is naturally covered by thorns. It was also used as a weapon: more than 30,000 palms were cut down to submit the natives to hunger [40]. The same chronicle attests to the consumption of hearts of palm by a *Chichimeca* army under the orders of the Spaniards. Before the Spanish invasion, the fruit of *B. gasipaes* was a staple, and the harvest was one of the most important events of the year, with most births taking place nine months after it. Even today, 500 years later, the use of the species has not reached back to its pre-Hispanic level [15,17,41]. *B. gasipaes* fruit has been relegated pejoratively as a “fruit of the Indians”, a forgotten, neglected fruit [39,42]; however, in times of scarcity it has been used to provide food security [43], and it still is today [9].

## 7. Traditional Uses

The main use of this species is timber. It is suggested that domestication of the species was primarily because of its wood [32]. The species is also an important part of the Amazonian and Central American foodways and ethnopharmacology, and material and social uses are also listed [44]. The main morphological structure from an ethnopharmacological perspective is the root, used to reduce inflammation and infections, and both to promote female fertility and act as an aid during pregnancy [20].

From the alimentary perspective, the main products are hearts of palm and fruits. The palm heart, already documented in the sixteenth century, is today one of the most important non-timber products in South America [45], and probably the best-known part of *B. gasipaes* worldwide, used in the preparation of salads, pizza, and even ceviche [46]. *B. gasipaes* hearts of palm are replacing those of other species, often endangered by overharvesting, mainly *Euterpe* spp. Being a cespitose species, the plant is not killed by prudent harvesting and thus a more sustainable product can be obtained. Canned hearts of palm are currently exported by several American countries. Ecuador and Bolivia are the largest exporters [47].

The fruit, despite not being produced in the same proportion, is a popular traditional food that needs to be processed prior to consumption, due to its high oxalate and other antinutrient content [48]: cooked fruit, flour, fermented *chicha*, slowly fermented silage [38], and oil are among the traditional uses. The fruit and its processing byproducts have also been used as animal and fish feed [5,49,50,51]. Larger fruit is less palatable than smaller fruit, due to a coarse, dry texture. To compensate, the fruit is frequently consumed with mayonnaise or sour cream.

An indirect alimentary use of the species is the use of the decomposing trunk after felling the palm to raise *chontacuros*, the larvae of *Rhynchophorus palmarum* L., a “…delicious, butter-tasting” delicacy [52] in the Ecuadorian and Peruvian Amazonia rich in protein, vitamin E, and minerals, also used against cough, asthma, and other respiratory affections [53,54,55].

Some Indigenous communities, such as the Colombian Uitoto, do not have access to table salt, so they make “bush salt,” obtained from burning, dissolving, filtering, and drying plant material, including the barkless stem of *B. gasipaes*. This process yields a mixture of salts: chloride, sulfate, and carbonate are the main anions, and potassium is the main cation. These salts are not particularly good tasting because of the high carbonate and low sodium content: their taste was described by Spanish chronicler Juan de Castellanos in the sixteenth century as “almost having the taste of sardines and herrings.” There is also a cosmic, alchemical, significance for communities, where extracting salt is considered to be the cleansing of the evil and disease and converting them into human food [56].

In other uses, the wood is used in the manufacture of flooring, marimbas, bows and arrows, spears, knives, and building material, the thorns are used as needles, the leaves for roofing, the fruit is used together with *Clibadium surinamense* L. leaves to make fish poison [53]. Table 2 summarizes traditional ethnomedical, alimentary, and other uses of *B. gasipaes*. The use of the wood is so present that the verb *achuntar* (to hit the mark) has entered the Spanish language from *chunta*, the quechua name *for B. gasipaes*, referring to the use of the wood to make arrows [57].

## 8. Modern Uses

Aside from the traditional uses, new applications are being found for *B. gasipaes*. Chonta pulp, in combination with other materials, is being implemented in the decontamination of water of minerals such as lead and cadmium [29,60]. Sensory evaluation and consumer acceptance analysis has been performed: oily fruit is more attractive and less averse than starchy fruit [61]. This may influence genetic development of the species and also guide the manufacture of food products from the fruit. Food products are being produced and marketed other than hearts of palm: jam, jelly, wine, flour and flour products (bread, pudding, pasta, etc.) [62,63].

There is a variety of alimentary and industrial applications and new uses for *B. gasipaes,* both as product and from the industry byproducts: functional food ingredients, brewing, dyes, and others. Some examples are listed in Table 3.

## 9. Nutritional Composition

The fruit of the peach palm, *macrocarpa* variety, is a starchy, ancestral staple, while *meso* and *microcarpa* varieties are used as snacks due to their smaller size, and to obtain oil. In order to consume the fruit, it is necessary to ferment or cook it for 1–3 h, usually in salt water, to remove the irritating oxalate crystals usually found in red drupes and reduce other antinutrients, such as phytates and tannins [20,71,72]. The nutritional composition of the fruit is summarized in Table 4 and that of the flour in Table 5.

The fruit of *B. gasipaes* is characterized by the amount of carbohydrates and fats contained that tend to increase when cooked. The oil obtained from the mesocarp is rich in unsaturated fatty acids, particularly oleic acid, while saturated fatty acids such as lauric acid are found in the seed [73,74]. The fruit is also high in dietary fiber, and although the protein content is low, all essential amino acids are present. Essential minerals are also present: K (12%), Se (9%), and Cr (9%) are the most abundant [5,24]. In addition, the bromatological characterization of the epicarp or peel shows: protein, 2.3%; ash, 2.3%; raw fiber, 8.2%; detergent acid fiber, 13.4%; and detergent neutral fiber, 63.6%. This composition supports the use as raw material for the production of flour [75,76].

The industrialization of products derived from this fruit has been increasing in recent years. The production of flour obtained from the mesocarp of the fruit, in combination with other flours such as wheat, corn, or quinoa and technological adjuvants, are acceptable by consumers and can be used in different matrices (sandwiches, cakes, pasta), the high carotenoid content makes the flour a potentially functional ingredient [66,77,78]. Nutritional composition of fruit from different origins is summarized in Table 4.

There is variability in the nutritional values that can be explained by the different *B. gasipaes* varieties, size, and starch–oil composition of the fruit. The nutritional parameters of flours from different origins are shown in Table 5. Flour from *macrocarpa* and seedless varieties show higher carbohydrate content. Additionally, flour from seedless fruits has a higher protein content.

The oil obtained from *B. gasipaes* is rich in mono and polyunsaturated fatty acids, particularly oleic, with the presence of ω-3 and ω-6 acids and bioactive lipids. It can be considered a heart-healthy oil [82,83].

## 10. Biological Activity

The information derived from research on the biological activity of the fruit of *B. gasipaes* (pulp, seed, peel, or whole fruit) has been summarized in Table 6—antioxidant activity, and Table 7—other biological activity. Most studies focus on antioxidant and protective activity, due to the presence of carotenoids. There are other studies on its biological activity that are not related to its traditional use. Within these activities we can mention the hypoglycemic capacity, modulation of lipid metabolism, cytotoxic activity, and its effect on sperm motility [84].

## 11. Phytochemical Composition

**Figure 3:**
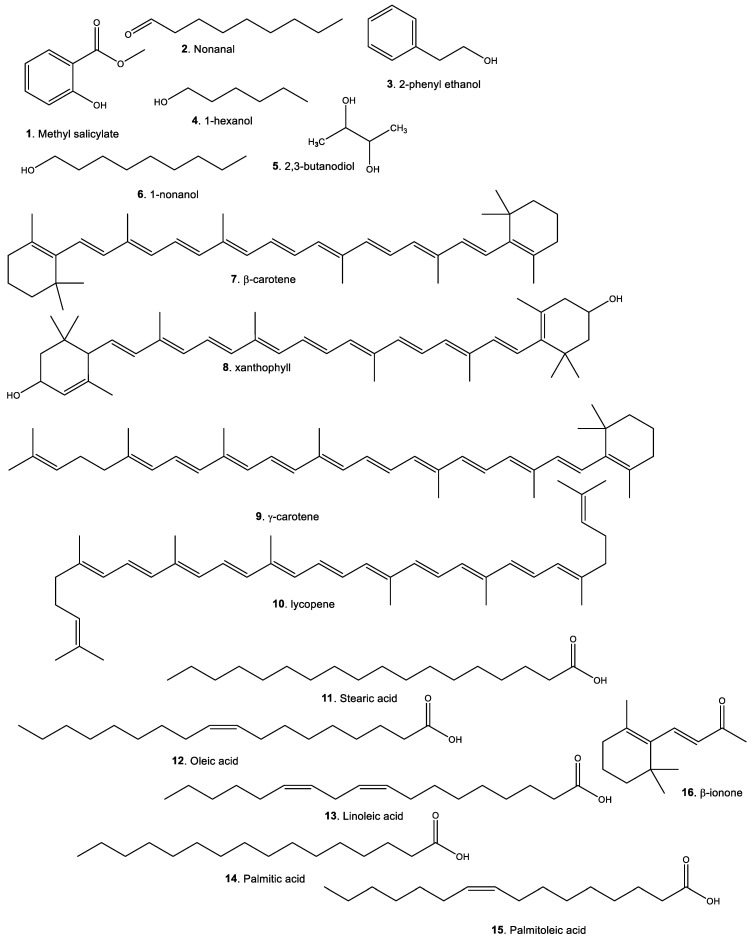
Phytochemical composition of *Bactris gasipaes* fruit. Major compounds.

According to the bibliographic data collected; the phytochemical composition of fruit pulp in concentrations greater than 1 mg/100 g, comprises 16 phytochemical compounds (Table 8 and Figure 3), which include: one ester, one aldehyde, four alcohols, four carotenoids, five fatty acids, and one aromatic compound. However, compounds in nanogram quantities have also been identified through ultra-high performance liquid chromatography coupled to tandem mass spectrometry with quadrupole analyzer (UHPLC–MS/MS), i.e., protocatechuic acid (30 ng/g), chlorogenic acid (20 ng/g), *p*-coumaric acid (16.6 ng/g), ferulic acid (160 ng/g). In addition, flavonoids such as myricetin (20 ng/g), apigenin (2.1 ng/g) [85], and also rutin, catechin [90], vicenin-2, and schaftosides [26].

The analysis carried out by [82] from the pulp oil analyzed by gas chromatography, identified minoritarian fatty acids: myristic acid (0.10%), margaric acid (0.11%), arachidonic acid (0.24%), and linolenic acid (1.17%). Fatty acid composition changes by variety [91]

In the comparisons among the lipid extraction from of pulp (2 g) of four varieties of Colombia, obtained by Soxhlet extraction using hexane and analyzed by gas chromatography (GC) with flame ionization detector, shows that the fatty acids present, respectively, are: lauric (0.014 and 0. 015%), myristic (0.12, 0.14, 0.13, and 0.10%), palmitic (34.9, 34, 39.9, and 34.5%), palmitoleic (7.9, 8.3, 9.5, and 10.8%), stearic (1.5, 1.6, 1.4, and 1%), oleic (51.9, 45.8, 38, and 46.4%), linoleic (2.4, 8, 8.6, and 5.3%), linolenic (0.2, 0.9, 1.5, and 0.9%), saturated fatty acids (36.8, 36.1, 41.7, and 36.2%), and polyunsaturated fatty acids (2.6, 8.9, 10.1, and 6.2%) [92].

Today there is an interest in the food industry in replacing synthetic dyes such as tartrazine (yellow-orange color) as additives due to adverse reactions related to hypersensitization to salicylic acid, complications in respiratory diseases, and long-term genotoxic effects [93] of dyes such as β-carotenes, although they are not as stable in extreme conditions [94,95]. However, carotenes have an added value due to the great diversity of biological activities they present, mainly as antioxidants. From the raw and cooked pulp, *α*-carotene, *E*-*γ*-carotene, and *E*-lycopene have been identified using high-resolution liquid phase chromatography with a diode network detector and visible ultraviolet spectroscopy (HPLC-DAD-UV/Vis) [86], and HPLC with a photodiode-array detector (PDA) coupled to mass spectrometry with an ion trap analyzer (MS) and ultraviolet spectroscopy (UV/Vis). The concentration of these components varies according to the place of origin of the sample, where the ones originating in Bolivia are those that presented higher concentrations [96,97], while Santos [98] have reported the identification of lutein (11.9 mg/Kg) and *cis* lutein (2.22 mg/Kg) using HPLC-PDA-UV/Vis.

A comparison of carotenoid extraction from pulp, between supercritical fluids, traditional methods of methanolic extraction, and the Soxhlet extraction method using petroleum ether was performed [99]. The highest extract yield was that obtained by Soxhlet extraction, followed by methanolic extraction, and finally supercritical fluids with extraction conditions of 300 bar and −60 °C. The concentration of carotenoids (mg/g of extraction) differs from the yield, with the order of highest to lowest extraction: Soxhlet, supercritical fluids, and methanolic extraction. However, the antioxidant activity evaluated by spectroscopic techniques compared to caffeic acid and quercetin show that the best antioxidant activity is that of the extract using the Soxhlet extraction, then methanolic extraction, and finally supercritical fluids, most likely the type of carotenoids obtained differs with the type of extraction.

Another promising method is ultrasound-assisted extraction, in this case to obtain total carotenoids from the peel of the chonta fruit and optimize extraction conditions: ultrasonic intensity (1528 W/m^2^), temperature (35 °C), and time (30 min), obtained 163.47 mg carotenoids per 100 g fruit [100], which is considered a significant amount of carotenoids.

## 12. Sustainable Development Goals (SDG)

*B. gasipaes* cultivation and uses are linked to several SDGs through food and non-food initiatives. Examples include farming, products recovered from waste, and food safety are shown in Table 9. The main SDGs are Responsible Consumption and Production (nine publications), Life on Land (five), and Climate action (three).

## 13. Major Concerns

Even though the species is widely spread, there is concern for the loss of populations due to soybean cultivation, forest clearance, and road building [109], as well as genetic erosion due to centuries of neglect [38]. Lately the forest fires due to anthropogenic action and climate change have risen as a concern. The modification of current cultivation areas, with an allover descent in surface due to the climate crisis [7] is also a major concern.

## 14. Conclusions

The peach palm is a very important species in tropical America, especially as a food source that not only provides food safety but also several opportunities for sustainable industrial production. The fruit is a rich source of bioactive compounds with significant antioxidant capacity, and nutritional, both macro and micronutrient, and functional properties.

Prudent cultivation and use of *B. gasipaes* in a way respectful of traditional methods, coupled with sustainable innovation has potential for advancing the SDGs.

Despite its widespread cultivation area, populations, and genetic diversity of the species, they are at risk due to deforestation, neglect, and the climate crisis.

## Figures and Tables

**Table 1:** Common names of *Bactris gasipaes* according to location.

Country	Names
Brazil	Papunha; Pupunha; Pupunheira; Popunha.
Bolivia	Chonta; Palma de Castilla; Tembe; Chima; Anua; Mue; Huanima; Pupuña; Tëmbi; Eat; Tempe.
Colombia	Cachipay; Chantaduro; Chenga; Chonta; Chontaduro; Chichagai; Pijiguay; Pupunha; Pupuña; Pejibá; Jijirre; Macanilla; Contaruro; Have; Pipire.
Costa Rica	Pejibaye; Pejivalle.
Ecuador	Chonta; Chonta dura; Chontaduro; Chonta palm; Palmito; Chantaduro, Puka chunta; Shalin chunta
Guyana	Paripie; Parepon.
Peru	Chonta; Ruru; Pejwao; Pifuayo; Chonta Duro; Joó; Uyai; Mee; Pijuayo; Pisho-Guayo; Sara-Pifuayo.
Suriname	Amana; Paripe; Paripoe.
Venezuela	Bobi; Cachipaes; Macana; Peach; Pijiguao; Pixabay; Rancanilla; Gachipaes; Pichiguao; Piriguao; Cachipay; Pachigaro.

Adapted from [16,19].

**Table 2:** Traditional uses of *B. gasipaes* ordered by plant organs.

Plant Organ	Applications	Ref.
**Ethnomedical**		
Fruit	Body and head aches, inflammation of eyes and gall bladder, galactagogue, infertility, cough, colds, psoriasis, tuberculosis.	[19]
Leaf	Ear pain, epilepsy, baths that prevent premature birth, energy cleansing.	[19]
Palm, heart of palm	Stomach, ear, menstrual and muscle pain, eye inflammation, inguinal hernia, malaria, chickenpox, hepatitis, childbirth problems, infertility, and uterine infections, anemia, antiophidic, prevention of baldness, galactagogue, sedative, hot hearts of palm are rubbed on children to dispel panic attacks.	[19,53]
Seed	Stomach pain, cancer	[19]
Root	Urinary and menstrual problems, inguinal hernia, uterine infections, colds and pneumonia, epilepsy, body, stomach, and ear pains, diarrhea (decoction), eye inflammation, vermicide, postpartum depression, galactagogue (washed with cooked root), contraceptive, prevention of baldness, hepatitis, malaria, mastitis, prevention of abortions, hemorrhages in childbirth, fertility, and aphrodisiac.	[19,53,58]
**Alimentary**		
Fruit	Cooked, roasted, dried, canned, jams and preserves, flour. Drinks: “chucula” (mixed with banana), “chicha” and juices. Oil for human consumption.	[19,53]
Palm	It is consumed preferentially cooked due to the antinutrient content: as a side dish, salad, filling, *ceviche*. Consumed dry or canned in brine.	[19,46,53,58]
Seed	Cooking oil, dried and ground to prepare a coffee-like drink, toasted as a snack.	[19,53]
**Other**		
Fruit	Cosmetic, fuel, and lubricating oil, fertilizer, hunting and fishing bait, dye, animal feed, fish poison mixed with *Clibadium surinamense* leaves.	[19,53,58]
Seed	Cosmetic and soapmaking oil, body adornment, toy (marbles), handicrafts.	[19]
Leaf	Body adornment, thatching, baskets, mats, and fan weaving, dying, wrapping, fuel, animal feed, paper, fertilizer.	[19,53,58]
Wood	Manufacture of marimbas, knives, blowguns, spears, good luck charms, vessels, bows, arrows, looms, pylons, macanas, walls and wall sidings, corrals, canoes, beds, weaving spindles, flooring (parquet), fishing rods and traps, ceremonial tables, and altars of healing, hollow trunk as a conduit or trough, manufacture of paper and fertilizers.	[53,58]
Thorn	Removal of other thorns, sewing needles, witchcraft.	[53,58,59]

**Table 3:** Modern uses for *B. gasipaes*.

Plant Organ	Use	Ref.
Fruit	Microwave assisted extraction, extract emulsified for improved carotenoid bioavailability.	[64]
Fruit	Fermentable substrate for lager beer brewing.	[65]
Peel	Natural food dye from peel flour.	[66]
Peel	Emulsified flavoring paste.	[67]
Peel, pulp	Functional food additives.	[68]
Plant	*B. gasipaes* industrial waste as substrate for xylanase production.	[69]
Plant	*B. gasipaes* and *Theobroma cacao* residues used as substrate for edible mushroom cultivation.	[70]
Pulp	In combination with other materials, heavy metal decontamination of water.	[29,60]

**Table 4:** Nutritional composition of *B. gasipaes fruit* from different origins.

Parameter (per 100 g Cooked Fruit)	Colombia [79]	Costa Rica [71]	Peru [80]	Brazil [63]	Brazil, White [25]
Energy (Cal)	358	185–196	184	-	266.6
Carbohydrates (%)	19.0	37.6–41.1	41.0	24.05–44.16	43.76 ± 1.76
Moisture (%)	48.2	50.5–52.2	52.3	47.98–63.96	28.85 ± 0.57
Lipids (%)	25.7	4.4–4.6	3.2	2.62–6.88	7.80 ± 1.85
Protein (%)	6.3	2.6–3.3	2.8	2.00–3.90	5 ± 1
Fiber (%)	12.7	1.0–1.4	1.3	-	10 ± 1
Calcium (mg)	81	14–23	23	21.8 ± 2.4	150 ± 18
Phosphorus (mg)	47	-	20	-	86 ± 12
Iron (mg)	0.7	0.7–1.0	0.65	-	2.3 ± 0.57

**Table 5:** Nutritional parameters, *B. gasipaes* flour.

Parameter (per 100 g Flour)	Microcarpa [63]	Mesocarpa [63]	Macrocarpa [63]	Seedless [81]
Carbohydrates (%)	67.32 ± 0.4	66.68 ± 0.92	75.02 ± 0.23	76.2 ± 0.8
Moisture (%)	12.21 ± 0.41	12.58 ± 0.51	9.60 ± 0.17	6.94 ± 0.04
Lipids (%)	7.40 ± 0.20	4.73 ± 0.04	3.95 ± 0.13	7.8 ± 0.7
Protein (%)	4.62 ± 0.03	3.20 ± 0.02	2.46 ± 0.02	6.9 ± 0.1
Fiber (%)	5.47 ± 0.70	10.82 ± 0.43	7.67 ± 0.18	
Energy (kcal)	300.07 ± 2.46	326.67 ± 2.01	321.28 ± 1.64	403 ± 2

**Table 6:** Antioxidant activity of *B. gasipaes*.

MS	Material	Model	Method	Result	Ref.
Pulp	Aqueous ethanolic extract	In vitro	ABTS	88 ± 1%; SC_50_ 30.5 ± 0.6 μg/mL CE	[85]
			H-ORAC_FL_	602 ± 20 μmol TE/g DE	
Dehydrated pulp	Hexane extract	In vitro	H-ORAC	45.56 ± 1.96 μmol TE/g of dehydrated fruit	[71]
Raw and cooked pulp	Varieties of countries	In vitro	DPPH (IC_50_)	Crude: Costa Rican variety 16.3 μg carotenoids/mL Cooked: Bolivian variety 24.1 μg carotenoids/mL	[86]

MS: Morphological structure. DPPH: 2,2-diphenyl-1-picrylhydrazyl. IC_50_: Maximum inhibitory concentration (50%). ABTS: 2,2 azino bis (3-ethylbenzothiazolin-6-sulfonic acid). CE: Crude extract. H-ORAC_FL_: Absorbance capacity of hydrophilic oxygen radicals–fluorescein. TE: Trolox equivalents. DE: Dry extract.

**Table 7:** Biological activity of *Bactris gasipaes*.

Activity	MS	Product/Model	Mechanism	Result	Ref.
Hypoglycemic	Pulp	In vivo Healthy individuals (18–51 years)	Post-prandial glucose measured at 30, 60, 90 and 120 min after ingesting 25 g of pulp.	Smaller Glycemic index than bread 80 ± 9 mg/dl-60 to 90 min	[87]
HDL increase	Pulverized fruit	Commercial rat food supplemented with red *B. gasipaes* fruit /Rats	Daily feeding with the mixture for 30 days.	Infant group: <weight gain and BMI, cholesterol and >HDL cholesterol Post-infant group: <triglycerides and >HDL cholesterol	[88]
Hypolipemiant	Pulp and seed	Fruit flour and lysine In vivo 16 pigs, fattening phase for 35 days	Percentage of flour (0, 16, and 32) and lysine (0 and 0.27) <lipid content Improves the properties of lean meat	Addition of flour in 16% and lysine 0.17%-meat with >dry matter content and moisture <	[89]
Cytotoxicity	Pulp	ETHOS In vitro MCR-5 cells	Viability of MCR-5 when exposing cells to concentrations of 6.25 to 200 μg/mL/72 h	Causes death in normal lung cells.	[85]
Effect on sperm motility	Pulverized pulp	Aqueous extract In vitro Sperm	Progressive agglutination of sperm Reduces its rectilinear velocity and linearity index.	Non-permanent action, thermolabile at 100 °C for 1 min	[84]
Liver protection	Pulp	Acetone extract In vivo 5 Sprague–Dawley rats	Liver tissue of each rat subjected to oxidative stress with HPTB and then measure the reactive substances to thiobarbituric acid—the final product of lipid peroxidation.	Hepatoprotective effect against oxidative stress <CI_50_ with a concentration of 10.9 μg carotenoids/mL	[87]

Note. The acronyms correspond to: SM: Morphological structure. min: Minutes. N: Number. BMI: body mass index. ETHOS: Microwave extraction with aqueous ethanol solution. MCR-5: A cell line of human lung fibroblasts. h: Hours.

**Table 8:** Phytochemical composition of the fruit of *Bactris gasipaes*.

No.	Compound	I	A	PO	Method	Reference
**Ester**						
1	Methyl salicylate	x		Raw pulp	ETHOS; HS-SPME/GC-MS	[85]
**Aldehyde**						
2	Nonanal			Raw pulp	ETHOS; HS-SPME/GC-MS	[85]
**Alcohols**						
3	2-phenylethanol	x		Raw pulp	ETHOS; HS-SPME/GC-MS	[85]
4	1-Hexanol	x		Raw pulp	ETHOS; HS-SPME/GC-MS	
5	2,3-Butanodiol	x		Raw pulp	ETHOS; HS-SPME/GC-MS	
6	1-Nonanol	x		Raw pulp	ETHOS; HS-SPME/GC-MS	
**Carotenoids**						
7	β-carotene	x		Raw and boiled pulp	HPLC-DAD-UV/Vis	[86]
8	Xanthophyll	x		Raw and boiled pulp	HPLC-DAD-UV/Vis	
9	γ-carotene	x		Raw and boiled pulp	HPLC-DAD-UV/Vis	
10	Lycopene	x		Raw and boiled pulp	HPLC-DAD-UV/Vis	
**Fatty acids**						
11	Stearic acid	x		Pulp (oil)	SOX; GC-MS	[82]
12	Oleic acid	x		Pulp (oil)	SOX; GC-MS	
13	Linoleic acid	x		Pulp (oil)	SOX; GC-MS	
14	Palmitic acid	x		Pulp (oil)	SOX; GC-MS	
15	Palmitoleic acid	x		Pulp (oil)	SOX; GC-MS	
**Other**						
16	β-ionone	x		Raw pulp	ETHOS; HS-SPME/GC-MS	[85]

Note. I: identified; A: isolated; PO: plant organ. Methods are indicated with an acronym, corresponding to: ETHOS: Microwave extraction with aqueous ethanol solution. HS-SPME/GC-MS: Microextraction in solid phase in headspace mode (HS-SPME) coupled to gas chromatography/mass spectrometer. HPLC-DAD-UV/Vis: high resolution liquid phase chromatography (HPLC) and visible ultraviolet spectroscopy (UV/Vis). SOX: Soxhlet (solid liquid) extraction method, using petroleum ether. GC-MS: gas chromatography coupled to mass spectrometry.

**Table 9:** Sustainable Development Goals (SDG) initiatives including *B. gasipaes*.

Initiative	SDG	Ref.
Agroforestry indigenous farming	1, 2, 13, 15	[101]
Recovery of biological pigments with alternative solvents	9	[102]
*B. gasipaes* recovered carotenoid supplements	9, 12	[103]
Production of antioxidant xylooligosaccharides from *B. gasipaes* waste	12	[104]
Ethanol production	12	[105]
Dietary diversity through underutilized species	1, 15	[106]
Materials for jewelry	13	[107]
Integration into circular economy	12	[108]

SDG: 1-No poverty; 2-Zero hunger; 9-Industry, innovation, and infrastructure; 12-Responsible consumption and production; 13-Climate action; 15-Life on land.

## Data Availability

Not applicable.

## References

[1] Aguirre Mendoza Z., León Abad N. (2011). Sobrevivencia y Crecimiento Inicial de Especies Vegetales En El Jardín Botánico de La Quinta El Padmi, Zamora Chinchipe. Arnaldoa.

[2] Clavijo J., Yánez P. (2017). Plantas Frecuentemente Utilizadas En Zonas Rurales de La Región Amazónica Centro Occidental de Ecuador. INNOVA Res. J..

[3] Brito B., Paredes N., Vargas Y., Caicedo C., Buitrón L., Díaz A., Velástegui F., Yánez C., Cuasapaz P. (2018). Calidad y Valor Agregado de Los Frutales Amazónicos. Proceedings of the Primer Congreso Internacional Alternativas Tecnológicas para la Producción Agropecuaria Sostenible en la Amazonía Ecuatoriana.

[4] Toledo D., Brito B. (2008). Aprovechamiento Del Potencial Nutritivo y Funcional de Algunas Frutas de La Amazonía Ecuatoriana.

[5] Vargas V., Clement C., Moraes M., Moraes M. (2020). Bactris Gasipaes (Arecaceae): Una Palmera Con Larga Historia de Aprovechamiento y Selección En Sud América. Palmeras y usos: Especies de Bolivia y la Región.

[6] Arunachalam V. (2012). Peach Palm. Genomics of Cultivated Palms.

[7] Pirovani D.B., Pezzopane J.E.M., Xavier A.C., Pezzopane J.R.M., de Jesus Júnior W.C., Machuca M.A.H., dos Santos G.M.A.D.A., da Silva S.F., de Almeida S.L.H., de Oliveira Peluzio T.M. (2018). Climate Change Impacts on the Aptitude Area of Forest Species. Ecol. Indic..

[8] Bush M.B., Nascimento M.N., Åkesson C.M., Cárdenes-Sandí G.M., Maezumi S.Y., Behling H., Correa-Metrio A., Church W., Huisman S.N., Kelly T. (2021). Widespread Reforestation before European Influence on Amazonia. Science.

[9] Graefe S., Dufour D., van Zonneveld M., Rodriguez F., Gonzalez A. (2013). Peach Palm (Bactris Gasipaes) in Tropical Latin America: Implications for Biodiversity Conservation, Natural Resource Management and Human Nutrition. Biodivers. Conserv..

[10] da Costa R.D.S., da Rodrigues A.M.C., da Silva L.H.M. (2022). The Fruit of Peach Palm (*Bactris Gasipaes*) and Its Technological Potential: An Overview. Food Sci. Technol..

[11] Vandermeer J., Aga A., Allgeier J., Badgley C., Baucom R., Blesh J., Shapiro L.F., Jones A.D., Hoey L., Jain M. (2018). Feeding Prometheus: An Interdisciplinary Approach for Solving the Global Food Crisis. Front. Sustain. Food Syst..

[12] Henderson A. (2000). Bactris (Palmae). Flora Neotrop..

[13] Govaerts R., Dransfield J. (2005). World Checklist of Palms.

[14] Buitrago Acosta M.C., Montúfar R., Guyot R., Mariac C., Tranbarger T.J., Restrepo S., Couvreur T.L.P. (2022). *Bactris Gasipaes* Kunth Var. *Gasipaes* Complete Plastome and Phylogenetic Analysis. Mitochondrial DNA Part B.

[15] Clement C., Araújo M., Coppens d’Eeckenbrugge G., dos Reis V., Lehnebach R., Picanço D. (2017). Origin and Dispersal of Domesticated Peach Palm. Front. Ecol. Evol..

[16] Lim T.K. (2012). Bactris Gasipaes. Edible Medicinal and Non-Medicinal Plants: Volumen 6, Fruits.

[17] Lehmann H., Gutmann O., Lasso M., Mayo R., Ponce T., Caicamo O., Silva V., Perez N., Riascos G., Muñoz M. (2013). Dramatic Fruit Fall of Peach Palm in Subsistence Agriculture in Colombia: Epidemiology, Cause and Control. Proceedings of the TROPENTAG 2013.

[18] Joas J., Le Blanc M., Beaumont C., Michels T. (2010). Physico-Chemical Analyses, Sensory Evaluation and Potential of Minimal Processing of Pejibaye (Bactris Gasipaes) Compared to Mascarenes Palms. J. Food Qual..

[19] Paniagua-Zambrana N.Y., Bussmann R.W., Romero C., Paniagua-Zambrana N.Y., Bussmann R.W. (2020). Bactris Gasipaes Kunth. Ethnobotany of the Andes.

[20] Paniagua N., Bussmann R., Romero C., Paniagua N., Bussmann R. (2020). Bactris Gasipaes Kunth.

[21] Gutiérrez C., Peralta R. (2001). Palmas Comunes de Pando.

[22] Ribeiro S.A., Coneglian R.C.C., Silva B.C., Deco T.A., Prudêncio E.R., Dias A. (2021). Shelf Life Extension of Peach Palm Heart Packed in Different Plastic Packages. Hortic. Bras..

[23] Stevanato N., Ribeiro T.H., Giombelli C., Cardoso T., Wojeicchowski J.P., Godoy E.D., Bolanho B. (2020). Effect of Canning on the Antioxidant Activity, Fiber Content, and Mechanical Properties of Different Parts of Peach Palm Heart. J. Food Process. Preserv..

[24] Theilkuhl S. (2018). Chontaduro Bactris Gasipaes (Kunth).

[25] dos Santos O.V., Soares S.D., Dias P.C.S., das Chagas Alves do Nascimento F., Vieira da Conceição L.R., da Costa R.S., da Silva Pena R. (2022). White Peach Palm (Pupunha) a New Bactris Gasipaes Kunt Variety from the Amazon: Nutritional Composition, Bioactive Lipid Profile, Thermogravimetric and Morphological Characteristics. J. Food Compos. Anal..

[26] Chisté R.C., Costa E.L.N., Monteiro S.F., Mercadante A.Z. (2021). Carotenoid and Phenolic Compound Profiles of Cooked Pulps of Orange and Yellow Peach Palm Fruits (Bactris Gasipaes) from the Brazilian Amazonia. J. Food Compos. Anal..

[27] Galluzzi G., Dufour D., Thomas E., van Zonneveld M., Escobar Salamanca A.F., Giraldo Toro A., Rivera A., Salazar Duque H., Suárez Baron H., Gallego G. (2015). An Integrated Hypothesis on the Domestication of Bactris Gasipaes. PLoS ONE.

[28] Cárdenas D., Marín N., Castaño N. (2012). Plantas Alimenticias No Convencionales En La Amazonía Colombiana y Anotaciones Sobre Otras Plantas Alimenticias. Rev. Colomb. Amaz..

[29] Vargas V., Moraes R. M., Roncal J. (2018). Fruit Morphology and Yield of Bactris Gasipaes in Tumupasa, Bolivia. Palms.

[30] Haro E.E., Szpunar J.A., Odeshi A.G. (2018). Dynamic and Ballistic Impact Behavior of Biocomposite Armors Made of HDPE Reinforced with Chonta Palm Wood (Bactris Gasipaes) Microparticles. Def. Technol..

[31] da Silva J., Clement C. (2005). Wild Pejibaye (Bactris Gasipaes Kunth Var. Chichagui) in Southeastern Amazonía. Acta Botánica Bras..

[32] Sosnowska J., Kujawska M. (2014). All Useful Plants Have Not Only Identities, but Stories: The Mythical Origins of the Peach Palm (*Bactris Gasipaes* Kunth) According to the Peruvian Asháninka. Trames J. Humanit. Soc. Sci..

[33] Herrera Angel L. (1975). Kanuma: Un Mito de Los Yukuna Matapí. Rev. Colomb. Antropol..

[34] Nantipia J.E., La Sánchez F. (1995). Celebración del rito de Uwi en el pueblo Shuar-Achuar (Fiesta de la Chonta). La Fiesta Religiosa Indigena en el Ecuador: Pueblos indigenas y educación.

[35] Villa Posse E. (1991). Mitos y Leyendas de Colombia: Selección de Textos.

[36] Nakashima D., Krupnik I., Rubis J.T. (2018). Indigenous Knowledge for Climate Change Assessment and Adaptation.

[37] Yucci T.E.V., León P., Torres D. (2017). Fiesta de la chonta y su impacto en el turismo comunitario del pueblo shuar. Kill. Soc..

[38] Mora-Urpí J., Hernández Bermejo J.E., León J. (1992). Pejibaye (Bactris gasipaes). Cultivos Marginados: Otra Perspectiva de 1492.

[39] Mora-Urpi J., Weber J.C., Clement C.R. (1997). Peach Palm: Bactris Gasipaes Kunth.

[40] Patiño V.M. (1960). Historia Colonial y Nombres Indígenas de La Palma Pijibay (Guilielma Gasipaes (HBK) Bailey). Rev. Colomb. Antropol..

[41] Prance G., Nesbitt M. (2005). The Cultural History of Plants.

[42] Popenoe W., Jimenez O. (1921). The Pejibaye a Neglected Food-Plant of Tropical America. J. Hered..

[43] Patiño V.M. (2002). Historia y Dispersión de los Frutales Nativos del Neotrópico.

[44] Rosete Blandariz S., Sáenz Véliz R.S., Jiménez González A., Pin Figueroa F.E., Rosete Blandariz S., Sáenz Véliz R.S., Jiménez González A., Pin Figueroa F.E. (2019). Fitorecursos de Interés Para El Turismo En Los Bosques Secos de La Región Costa, Jipijapa, Manabí, Ecuador. Rev. Cuba. Cienc. For..

[45] Brokamp G. (2015). Relevance and Sustainability of Wild Plant Collection in NW South America.

[46] Duarte-Casar R., Robalino-Vallejo J., Buzetta-Ricaurte M.F., Rojas-Le-Fort M. (2022). Toward a Characterization of Ecuadorian Ceviche: Much More than Shrimp. J. Ethn. Foods.

[47] World Bank; World Trade Organization Palm Hearts; Prepared or Preserved, Whether or Not Containing Added Sugar, Other Sweetening Matter or Spirit Exports by Country |2021. https://wits.worldbank.org/trade/comtrade/en/country/ALL/year/2021/tradeflow/Exports/partner/WLD/product/200891.

[48] Bolanho B.C., Danesi E.D.G., Beléia A.P. (2013). Peach Palm (Bactris Gasipaes Kunth) Characterization and the Potential of by-Products Flour Processing. Food Sci. Technol. Res..

[49] Abril R., Aguinda J., Ruiz T., Alonso J. (2015). Plant Species Used in Animal Feeding in Mera, Santa Clara and Pastaza Cantons in Pastaza Province, Ecuador. Rev. Cuba. Cienc. Agríc..

[50] Colina J., Méndez A., Araque H., Rueda E., León M., Rossini M. (2011). Lípidos Sanguíneos En Cerdos Alimentados Con Pijiguao (Bactris Gasipaes Kunth) y Lisina Sintética. Rev. MVZ Córdoba.

[51] Mosquera Perea D., Martínez Guardia M., Medina H., Hinestroza L. (2013). Caracterización Bromatológica de Especies y Subproductos Vegetales En El Trópico Húmedo de Colombia. Acta Agronómica.

[52] Valarezo S.J.A. (2002). La Selva, Los Pueblos, Su Historia: Mitos, Leyendas, Tradiciones y Fauna de La Amazonía Ecuatoriana.

[53] V de la Torre L., Navarrete H., Muriel P., Macía M., Balslev H. (2008). Enciclopedia de Las Plantas Útiles Del Ecuador.

[54] Cartay R. (2018). Entre El Asombro y El Asco: El Consumo de Insectos En La Cuenca Amazónica. El Caso Del *Rhynchophorus Palmarum*(Coleoptera Curculionidae). Rev. Colomb. Antropol..

[55] Jaramillo-Vivanco T., Balslev H., Montúfar R., Cámara R.M., Giampieri F., Battino M., Cámara M., Alvarez-Suarez J.M. (2022). Three Amazonian Palms as Underestimated and Little-Known Sources of Nutrients, Bioactive Compounds and Edible Insects. Food Chem..

[56] Echeverri J.Á., Román Jitdutjaaño Ó., Román S., Franky Calvo C.E., Zárate C.G., Franco F. (2001). Sal de Monte: Un Ensayo de ‘Halofitogenografía’ Uitoto. Imani mundo-estudios en la Amazonia Colombiana.

[57] Asale R. RAE achuntar | Diccionario de la Lengua Española. https://dle.rae.es/achuntar.

[58] Lim T.K. (2012). Edible Medicinal and Non-Medicinal Plants. Volume 1, Fruits.

[59] Chirif A. (2016). Diccionario Amazónico: Voces Del Castellano En La Selva Peruana.

[60] Pineda E., Guaya D., Tituana C., Osorio F., García M. (2020). Biochar from Agricultural By-Products for the Removal of Lead and Cadmium from Drinking Water. Water Switz..

[61] Martínez J.M., Moreno-Caicedo L.P., Loaiza-Loaiza O.A. (2021). Sensory Dimensions of Peach-Palm Fruit (Bactris Gasipaes) and Implications for Future Genetics. Agron. Mesoam..

[62] De Oliveira M., Martinez H., De Andrade J., Garnica M.G., Chang Y. (2006). Use of Pejibaye Flour (Bactris Gasipaes Kunth) in the Production of Food Pastas. Int. J. Food Sci. Technol..

[63] Pires M.B., Amante E.R., Lopes A.S., da Cruz Rodrigues A.M., da Silva L.H.M. (2019). Peach Palm Flour (Bactris Gasipae KUNTH): Potential Application in the Food Industry. Food Sci. Technol..

[64] de Souza Mesquita L.M., Neves B.V., Pisani L.P., de Rosso V.V. (2020). Mayonnaise as a Model Food for Improving the Bioaccessibility of Carotenoids from Bactris Gasipaes Fruits. LWT.

[65] de Souza P.G., Pantoja L., dos Santos A.S., Marinho H.A., de Almeida e Silva J.B. (2022). Avaliação Físico-Química de Cervejas Lagers Com Pupunha (Bactris Gasipaes) Em Bioprocessos Com Leveduras Livres e Imobilizadas. Conjecturas.

[66] Martínez J., Figueroa A.M., Ordóñez L.E. (2017). Effect of the Addition of Peach Palm (Bactris Gasipaes) Peel Flour on the Color and Sensory Properties of Cakes. Food Sci. Technol..

[67] Henao J.G., Mancheno G.G., Patiño A., Peña E.E., Páez M.I. (2021). Elaboración de una Pasta Emulsionada de Cáscara de Chontaduro (Bactris gasipaes). Rev. Tecnológica ESPOL.

[68] Amorim I.S., Almeida M.C.S., Chaves R.P.F., Chisté R.C. (2022). Technological Applications and Color Stability of Carotenoids Extracted from Selected Amazonian Fruits. Food Sci. Technol..

[69] Carvalho E.A., Nunes L.V., dos Santos Goes L.M., da Silva E.G.P., Franco M., Gross E., Uetanabaro A.P.T., da Costa A.M. (2018). Peach-Palm ( *Bactris Gasipaes* Kunth.) Waste as Substrate for Xylanase Production by *Trichoderma Stromaticum* AM7. Chem. Eng. Commun..

[70] Geni A.C., Tizá T.S., Camila O.B., da Costa Andréa M., Cid E.M.P., Antonio F.R.F., de Cássia Soares da Silva M., da Luz José M.R., Maria C.M.K., Givaldo R.N. (2022). Alkalinization and Moist Heat Treatments of Substrates for Cultivation of Edible Mushrooms in Pupunha and Cocoa Residues. Afr. J. Agric. Res..

[71] Madrigal G., Vargas R., Carazo G., Ramírez N., Baltodano E., Blanco J., Porras M. (2019). Phytochemical Characterization of Extracts of the Mesocarp of Bactris Gasipaes and Evaluation of Its Antioxidant Power for Pharmaceutical Dermal Formulations. Int. J. Herb. Med..

[72] Álvarez L. (2015). Plantas Promisorias de Uso Alimenticio Del Darién, Caribe Colombiano. Bol. Antropol. Univ. Antioquia.

[73] Valencia R., Montúfar R., Navarrete H., Balslev H. (2013). Plantas Ecuatorianas: Biología y Uso Sostenible.

[74] Correal C., Zuluaga G., Madrigal L., Caicedo S., Plotkin M. (2009). Ingano Traditional Food and Health: Phase 1, 2004–2005. Indigenous Peoples’ Food Systems: The Many Dimensions of Culture, Diversity and Environment for Nutrition and Health.

[75] Martínez J., Rodríguez X., Pinzón L., Ordóñez L. (2017). Caracterización Fisicoquímica de Harina de Residuos Del Fruto de Chontaduro (Bactris Gasipaes Kunth, Arecaceae) Obtenida Por Secado Convectivo. Corpoica Cienc. Tecnol. Agropecu..

[76] Medina H., Martínez M., Bonilla J. (2007). Caracterización Bromatológica de Materias Primas y Subproductos En El Municipio de Quibdó, Chocó. Rev. Inst. Univ. Tecnológica Chocó Investig. Biodivers. Desarro..

[77] Dussán S., De la Cruz R., Godoy S. (2019). Estudio Del Perfil de Aminoácidos y Análisis Proximal de Pastas Secas Extruidas a Base de Harina de Quinua y Harina de Chontaduro. Inf. Tecnológica.

[78] López R., Pérez A., Ivankovich C., Calderón S., Pineda M. (2015). Evaluación de La Aceptación Por Consumidores de Un Bocadillo de Pejibaye (Bactris Gasipaes) y Estudio de Su Potencial Como Alimento Funcional. Arch. Latinoam. Nutr..

[79] Solano J., Instituto Colombiano de Bienestar Familiar (2018). Tabla de Composición de Alimentos Colombianos (TCAC).

[80] Reyes M., Gómez I., Espinoza C. (2017). Tablas Peruanas de Composición de Alimentos.

[81] Silva Ribeiro G., Conceição Monteiro M.K., Rodrigues do Carmo J., da Silva Pena R., Campos Chisté R. (2021). Peach Palm Flour: Production, Hygroscopic Behaviour and Application in Cookies. Heliyon.

[82] dos Santos O.V., Soares S.D., Dias P.C.S., de Paula de Almeida Duarte S., dos Santos M.P.L., das Chagas Alves do Nascimento F. (2020). Chromatographic Profile and Bioactive Compounds Found in the Composition of Pupunha Oil (Bactris Gasipaes Kunth): Implications for Human Health. Rev. Nutr..

[83] Vasconcelos dos Santos O., Medeiros Pereira G., Lima dos Santos M.P., Carvalho do Rosário R., Galvão Martins M., Das Chagas Alves do Nascimento F., Dias Soares S., Vieira da Conceição L.R. (2022). Potencial Nutricional e Funcional Do Óleo Da Pupunha Variedade Amarela (Bactris Gasipaes Kunth). Sci. Plena.

[84] Angulo R., Medina L., Valencia V., Cardona W. (2017). In Vitro Effect of Three Aphrodisiac Plants on Human Sperm Motility. Rev. Cuba. Plantas Med..

[85] Faria J., Valido I., Paz W., da Silva F., de Souza A., Acho L., Lima E., Boleti A., Marinho J., Salvador M. (2021). Comparative Evaluation of Chemical Composition and Biological Activities of Tropical Fruits Consumed in Manaus, Central Amazonia, Brazil. Food Res. Int..

[86] Jatunov S., Quesada S., Díaz C., Murillo E. (2010). Carotenoid Composition and Antioxidant Activity of the Raw and Boiled Fruit Mesocarp of Six Varieties of Bactris Gasipaes. Arch. Latinoam. Nutr..

[87] Quesada S., Azofeifa G., Jatunov S., Jiménez G., Navarro L., Gómez G. (2011). Carotenoids Composition, Antioxidant Activity and Glycemic Index of Two Varieties of Bactris Gasipaes. Emir. J. Food Agric..

[88] Piccolotto R., Gonzaga J., Souza R., Gassen M., Nascimento C., Bayona M., Marcon J., Barros J. (2015). The Consumption of Red Pupunha (Bactris Gasipaes Kunth) Increases HDL Cholesterol and Reduces Weight Gain of Lactating and Post-Lactating Wistar Rats. J. Aging Res. Clin. Pract..

[89] Jerez N., Colina J., Araque H., Jiménez P., Velazco M., Colmenares C. (2011). Composición Proximal y Contenido de Lípidos y Colesterol de La Carne de Cerdos Alimentados Con Harina de Pijiguao (Bactris Gasipaes Kunth) y Lisina Sintética. Arch. Latinoam. Nutr..

[90] Peixoto Araujo N.M., Arruda H.S., Marques D.R.P., de Oliveira W.Q., Pereira G.A., Pastore G.M. (2021). Functional and Nutritional Properties of Selected Amazon Fruits: A Review. Food Res. Int..

[91] Soares S.D., Santos O.V.D., Nascimento F.D.C.A.D., Pena R. (2022). da S. A Review of the Nutritional Properties of Different Varieties and Byproducts of Peach Palm (*Bactris gasipaes*) and Their Potential as Functional Foods. Int. J. Food Prop..

[92] Restrepo J., Vinasco L., Estupiñán J. (2013). Estudio Comparativo Del Contenido de Ácidos Grasos En 4 Variedades de Chontaduro (Bactris Gasipaes) de La Región Del Pacífico Colombiano. Rev. Cienc..

[93] Arroyave J., Garcés L., Arango Á., Agudelo C. (2008). La Tartrazina, Un Colorante de La Industria Agroalimentaria, Degradado Mediante Procesos de Oxidación Avanzada. Rev. Lasallista Investig..

[94] Restrepo M., Acosta E., Ocampo J., Morales C. (2006). Sustitución de Tartrazina Por Betacaroteno En La Elaboración de Bebidas No Alcohólicas. Rev. Lasallista Investig..

[95] Dalgo V., Rodríguez A., Brito H. (2020). Obtención Del Colorante Natural de La Bactris Gasipaes. Rev. Científica Dominio Las Cienc..

[96] Hempel J., Amrehn E., Quesada S., Esquivel P., Jiménez V., Heller A., Carle R., Schweiggert R. (2014). Lipid-Dissolved γ-Carotene, β-Carotene, and Lycopene in Globular Chromoplasts of Peach Palm (Bactris Gasipaes Kunth) Fruits. Planta.

[97] De Rosso V., Mercadante A. (2007). Identification and Quantification of Carotenoids, by HPLC-PDA-MS/MS, from Amazonian Fruits. J. Agric. Food Chem..

[98] Santos M., Alves R., Roca M. (2015). Carotenoid Composition in Oils Obtained from Palm Fruits from the Brazilian Amazon. Grasas Aceites.

[99] Espinosa F., Martinez J., Martinez H. (2014). Extraction of Bioactive Compounds from Peach Palm Pulp (Bactris Gasipaes) Using Supercritical CO2. J. Supercrit. Fluids.

[100] Ordóñez L., Pinzón L., González L. (2015). Optimization of Ultrasonic-Assisted Extraction of Total Carotenoids from Peach Palm Fruit (Bactris Gasipaes) by-Products with Sunflower Oil Using Response Surface Methodology. Ultrason. Sonochem..

[101] Heredia-R M., Torres B., Cayambe J., Ramos N., Luna M., Diaz-Ambrona C.G.H. (2020). Sustainability Assessment of Smallholder Agroforestry Indigenous Farming in the Amazon: A Case Study of Ecuadorian Kichwas. Agronomy.

[102] Souza Mesquita L.M., Martins M., Pisani L.P., Ventura S.P.M., Rosso V.V. (2021). Insights on the Use of Alternative Solvents and Technologies to Recover Bio-based Food Pigments. Compr. Rev. Food Sci. Food Saf..

[103] Santamarina A.B., de Souza Mesquita L.M., Casagrande B.P., Sertorio M.N., Vitor de Souza D., Mennitti L.V., Ribeiro D.A., Estadella D., Ventura S.P.M., de Rosso V.V. (2022). Supplementation of Carotenoids from Peach Palm Waste (Bactris Gasipaes) Obtained with an Ionic Liquid Mediated Process Displays Kidney Anti-Inflammatory and Antioxidant Outcomes. Food Chem. X.

[104] Vieira T.F., Corrêa R.C.G., de Fatima Peralta Muniz Moreira R., Peralta R.A., de Lima E.A., Helm C.V., Garcia J.A.A., Bracht A., Peralta R.M. (2021). Valorization of Peach Palm (Bactris Gasipaes Kunth) Waste: Production of Antioxidant Xylooligosaccharides. Waste Biomass Valorization.

[105] Fernandes F., Farias A., Carneiro L., Santos R., Torres D., Silva J., Souza J., Souza É., Post-Graduate Program in Biotechnology and Natural Resources of the Amazon, Amazonas State University, Manaus, Amazonas, Brazil, School of Technology, Amazonas State University, Manaus, Amazonas, Brazil (2021). Dilute Acid Hydrolysis of Wastes of Fruits from Amazon for Ethanol Production. AIMS Bioeng..

[106] Moura de Oliveira Beltrame D., Neves Soares Oliveira C., Borelli T., Andrade Cardoso Santiago R., Coradin L., Hunter D. (2018). Brazilian Underutilised Species to Promote Dietary Diversity, Local Food Procurement, and Biodiversity Conservation: A Food Composition Gap Analysis. Lancet Planet. Health.

[107] Pinho Pinheiro A.P., Moraes d’Almeida J.R. (2020). Peach Palm: Pseudo-Wood for Sustainable Jewelry Design. Mater. Today Proc..

[108] de C. Spacki K., Vieira T.F., Helm C.V., de Lima E.A., Bracht A., Peralta R.M. (2021). Pupunha (Bactris Gasipaes Kunth): Uma Revisão. Agricultura e Agroindústria no Contexto do Desenvolvimento Rural Sustentável.

[109] Clement C.R., Santos R.P., Desmouliere S.J.M., Ferreira E.J.L., Neto J.T.F. (2009). Ecological Adaptation of Wild Peach Palm, Its In Situ Conservation and Deforestation-Mediated Extinction in Southern Brazilian Amazonia. PLoS ONE.

